# Supplementary material for: Metabolic Effects of Anti-TNF-α Treatment in Rheumatoid Arthritis
Source: Diseases. 2023 Nov 9;11(4):164. doi: 10.3390/diseases11040164 (PMC10660495; doi:10.3390/diseases11040164)
Supplement: Supplementary file 1 [file diseases-11-00164-s001.zip › diseases-2634889-supplementary.pdf]

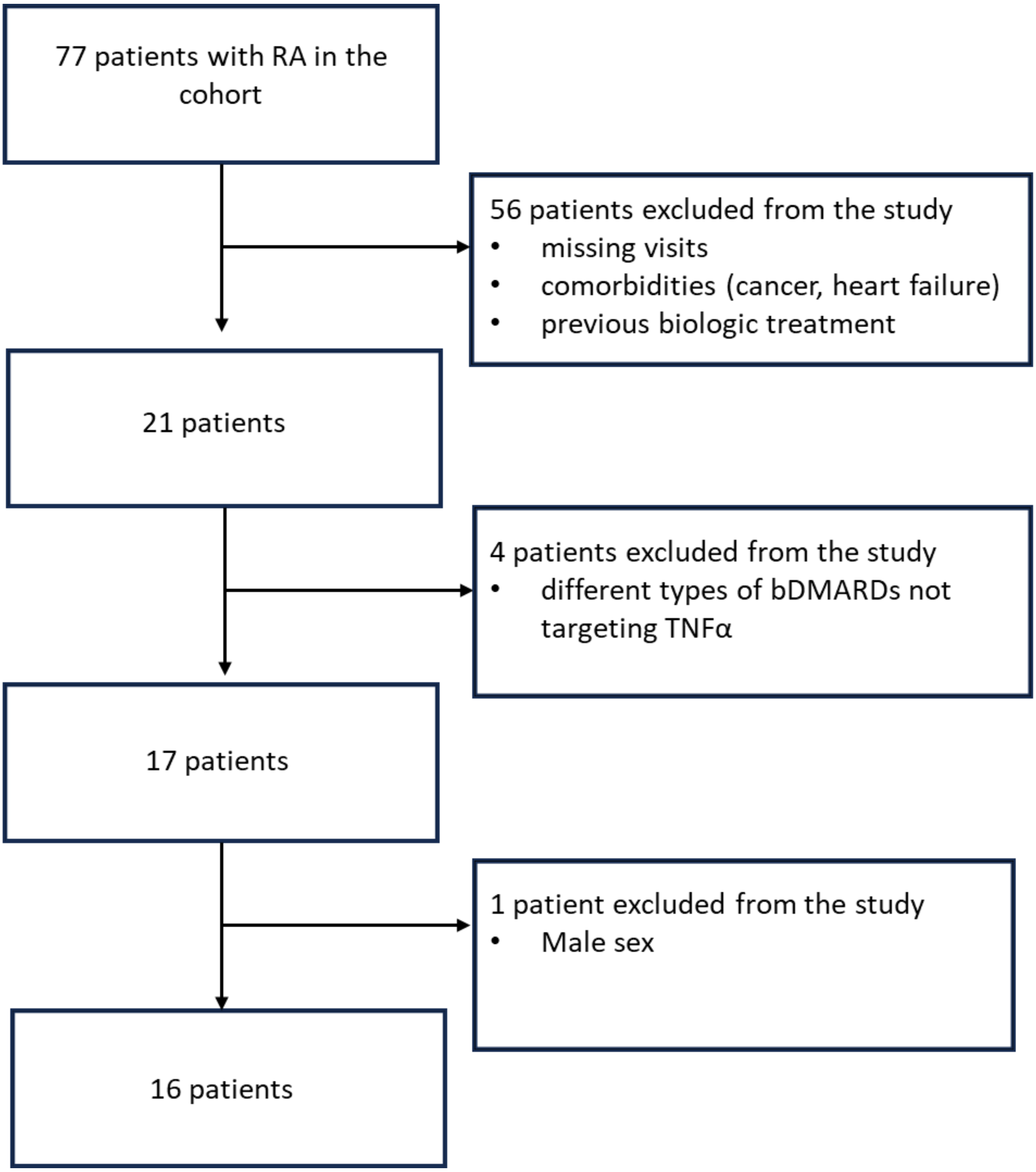

Supplementary Figure S1. Flow chart of patients after the application of the exclusion criteria
